# A Pharmacokinetic Study of Different Quercetin Formulations in Healthy Participants: A Diet-Controlled, Crossover, Single- and Multiple-Dose Pilot Study

**DOI:** 10.1155/2023/9727539

**Published:** 2023-08-10

**Authors:** Julia Solnier, Yiming Zhang, Kyle Roh, Yun Chai Kuo, Min Du, Simon Wood, Mary Hardy, Roland J. Gahler, Chuck Chang

**Affiliations:** ^1^ISURA, Burnaby, BC V3N4S9, Canada; ^2^School of Public Health, Faculty of Health Sciences, Curtin University, Perth, WA 6845, Australia; ^3^InovoBiologic Inc., Calgary, AB Y2N4Y7, Canada; ^4^Food, Nutrition and Health Program, University of British Columbia, Vancouver, BC V6T1Z4, Canada; ^5^Association of Integrative and Holistic Medicine, San Diego, California, USA; ^6^Factors Group R & D, Burnaby, BC V3N4S9, Canada

## Abstract

This study aimed to evaluate the blood concentrations of quercetin in healthy participants after the administration of different formulations in single- and multiple-dose phases. Ten healthy adults (males, 5; females, 5; age 37 ± 11 years) participated in a diet-controlled, crossover pilot study. Participants received three different doses (250 mg, 500 mg, or 1000 mg) of quercetin aglycone orally. In the single-dose study, blood concentrations (AUC_0–24_ and *C*_max_) of standard quercetin were compared with those of LipoMicel®—a food-grade delivery form of quercetin. In the multiple-dose study, blood concentrations of formulated quercetin were observed over 72 h, after repeated doses of LipoMicel (LM) treatments. The AUC_0–24_ ranged from 77.3 to 1128.9 ng·h/ml: LM significantly increased blood concentrations of quercetin by 7-fold (LM 500) compared to standard quercetin, when tested at the same dose, over 24 h (*p* < 0.001); LM administered at a higher dose (LM 1000) achieved 15-fold higher absorption (*p* < 0.001); LM tested at half a dose of standard quercetin increased concentration by approx. 3-fold (LM 250). Quercetin blood concentrations were attained over 72 h. The major metabolites measured in the blood were methylated, sulfate, and glutathione (GSH) conjugates of quercetin. Significant differences in concentrations between quercetin conjugates (sulfate vs. methyl vs. GSH) were observed (*p* < 0.001). Data obtained from this study suggest that supplementation with LipoMicel® is a promising strategy to increase the absorption of quercetin and its health-promoting effects in humans. However, due to the low sample size in this pilot study, further research is still warranted to confirm the observations in larger populations. This trial is registered with NCT05611827.

## 1. Introduction

Quercetin has been an important part of the human diet for centuries, and it can be found in various kinds of vegetables and fruits including apples, berries, black tea, buckwheat, cherries, citrus fruits, grapes, kale, onions, red wine, and tomatoes [1, 2], generally in its glycoside form [3]. In particular, the 3-OH group of quercetin represents a common glycosylation site—with quercetin 3-*O*-rutinoside (also known as rutin) as one of the most widespread forms of quercetin [4, 5].

In general, flavonols and flavones have shown protective effects against cancer and cardiovascular diseases in epidemiological studies [6, 7]. Specifically, quercetin has shown promising results *in vitro* and *in vivo* against cardiovascular diseases, cancer [8, 9], diabetes, allergy, asthma, and other atopic diseases [10]. Additionally, recent studies have also suggested that quercetin may have therapeutic effects as an adjuvant in COVID-19 infections [11–13]. Therefore, the absorption and bioavailability of quercetin may appear to be an important factor for bioefficacy.

Quercetin-conjugated glycosides have shown to affect the bioavailability, whereas the size and polarity of the compounds seemed to be decisive for crossing intestinal membranes [14, 15]. Other factors such as the food matrix, heat, pH, or metal ions during food processing as storage can affect the chemical stability (e.g., oxidation and degradation) and uptake of quercetin from foods [16].

From a nutritional perspective, the term bioavailability includes the extent of digestion, absorption, metabolism, and excretion of a compound after the ingestion of food [17]. Quercetin digested in the human body undergoes rapid metabolism (e.g., in the small intestine, liver, and kidneys) which involves glucuronidation, sulfation, and/or methylation [18]. The major absorption site of ingested quercetin is the small intestine, with only minor amounts absorbed in the stomach. Novel delivery systems can facilitate the penetration of a bioactive compound across the mucosal membranes while avoiding rapid metabolic modifications/or enzymatic degradation, which would ultimately result in low absorption. Common materials used in delivery systems to improve the bioavailability of various compounds include polymers like chitosan, cyclodextrins, and dendrimers [17].

Much research has been dedicated to the design of innovative technologies to improve the solubility and absorption of bioactive natural compounds [17, 19–21]. Thereby, the encapsulation of a compound into specific vehicles appears to be an encouraging method for shielding the substrate from undesirable conditions such as chemical or physical degradation and oxidation, and it can even help to mask unpleasant flavours [19, 20, 22]. Approaches that have been evaluated include micelles, colloidal systems, and use of nanoparticles. While nanoparticles have strong mobility through the GI tract, high potential drug-loading capacities, and high surface area to volume ratios [20], they have low internalization efficiency compared to lipid-based carriers due to the lack of specificity to intestinal lipoprotein transporters [23]. On the other hand, vectors with lipid-based carriers, complexes, micelles, and/or conjugates-based encapsulation demonstrate powerful promise to improve a compound's bioavailability and potentially boost clinical efficacy.

This study investigated the pharmacokinetic profile of a food-grade delivery formulation of quercetin (LipoMicel®, LM), which was compared against a standard formulation (control) and tested at three different doses in healthy participants. The pharmacokinetics of the different treatments were observed over 24 h after a single orally administered dose of quercetin (using AUC_0–24_ and *C*_max_). In addition, blood concentrations of quercetin were monitored over a 72 h period following multiple orally administered doses of formulated quercetin (LM) treatments (250 mg–1000 mg), and circulating metabolites of quercetin were also determined in human blood. The hypothesis of this study is that LM produces higher blood concentrations of quercetin than a standard/regular quercetin and that LM can sustain high blood concentrations of quercetin over a time period of 72 h. This new delivery system could have a wide range of applications from cancer treatments to nutraceutical, cosmeceutical, and other diagnostic purposes [24].

## 2. Materials and Methods

### 2.1. Participants

Ten healthy adults (5 men; 5 women; age 37.1 ± 11.2 years) with a body mass index (BMI) of 22.3 ± 2.5 kg/m^2^ and weight of 62.4 ± 11.2 kg were recruited (Table 1). Data are expressed as mean ± standard deviation (SD). Healthy participants who were ≥21 years old and of good physical condition—non-smokers and not taking any prescribed medication—were included in the trial.

Inclusion criteria included a signed written informed consent form and a willingness to avoid the consumption of any food and supplements that contain quercetin (48 h before each treatment and during the respective treatment periods).

As exclusion criteria, participants must not have any of the following diseases and/or health conditions: serious acute or chronic diseases—such as liver, kidney, or gastrointestinal diseases—which may affect absorption, metabolism, and/or elimination of the treatment, as well as any kind of contraindication and/or allergy to quercetin. Female participants must not have been pregnant, be planning to get pregnant, or be breastfeeding. Participants had to complete an online health questionnaire on their medical history upon study enrollment.

There was no control for the menstrual cycle phases of female participants. Thus, the potential impact of hormonal changes on drug absorption was not investigated in this study.

### 2.2. Treatments

The study was conducted with two different preparations of quercetin, procured from commercial sources (Natural Factors, BC, Canada). Each product in this study was administered (one or more capsules) as a single dose or as multiple doses at total doses of 250 mg, 500 mg, or 1000 mg quercetin aglycone, along with a glass of water (∼200 mL).

#### 2.2.1. The Standard Product

One hard gel capsule contained 250 mg quercetin, as well as carbohydrate gum, purified water, microcrystalline cellulose, stearic acid, magnesium stearate, and silica.

#### 2.2.2. The Investigational Product Quercetin LipoMicel®

One soft gel capsule contained 250 mg quercetin, as well as gelatin, glycerin, purified water, carob powder, medium chain triglycerides, xylitol, methylsulfonylmethane, *Stevia rebaudiana* leaf, peppermint essence, and lecithin. Soft gels were composed of quercetin encapsulated within a gelatin shell. Its micellar membrane contained food-grade excipients (patent pending).

All capsules were stored at room temperature, protected from heat, moisture, and direct light. Standard quercetin was encapsulated in a hard gelatin capsule; Quercetin LipoMicel was encapsulated in a soft gelatin capsule. Quercetin concentrations in the capsules were verified by Ultra-High-Performance Liquid Chromatography (UHPLC), and the concentrations were found to deviate less than 10% from their label claims. Both the standard product and Quercetin LipoMicel met USP<2040> requirements for disintegration. The dosage forms disintegrate or rupture within 30 minutes under test conditions.

### 2.3. Study Design

An open-label, crossover, pharmacokinetic study was performed with different quercetin formulations at the ISURA research facility (Burnaby, BC, Canada).

There was no placebo control group as each individual acted as their own control.

**Figure 1:**
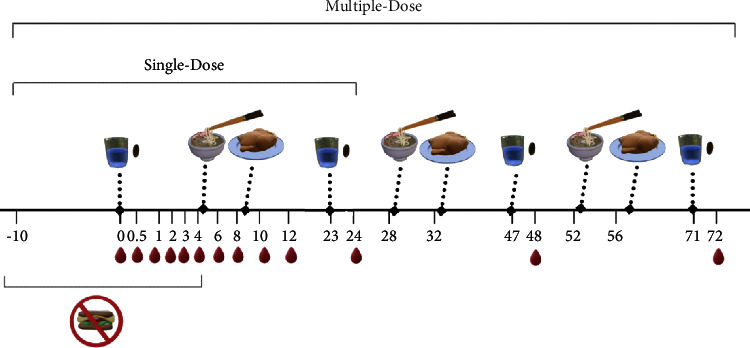
Study diagram illustrating the process of administrating the interventions: the standardized foods and the blood sampling times in the single-dose and multiple-dose phase. Participants are asked to fast from 10 h before taking the first intervention until after the 4 h blood sample has been taken.

The study included two phases conducted with the same ten healthy participants: a single-dose phase (24 h) and a multiple-dose phase (72 h). In the single-dose phase, there were four interventions: 250, 500, and 1000 mg of quercetin aglycone provided by Quercetin LipoMicel, as well as 500 mg of quercetin aglycone provided by the standard product. In the multiple-dose phase, the same four interventions were repeated daily for 72 h. Each phase also contained numerous time points at which the participants collected capillary whole blood samples (50 *μ*L per sample) via fingerpricking (Figure 1). Blood collection was conducted at the lab facility of ISURA (Burnaby, BC, Canada) under the supervision of qualified personnel for up to 8 h after product administration. Supervision continued at 24 h and 72 h post-administration, as necessary. After collection, each blood sample was deposited into a 1.5 mL microcentrifuge tube containing 5 *μ*L of 10% *w*/*w* ascorbic acid in water, and the tube was immediately closed and kept frozen at −20°C until further processing. Blood samples were analyzed by Liquid Chromatography Mass Spectrometry (LC-MS) within 24 h after processing.

Participants followed a washout period of at least 7 days between each treatment. All adverse events reported by participants were recorded throughout the study period.

The study protocol was approved by the Canadian SHIELD Ethics Review Board (Ontario, Canada) (OHRP Registration IORG0003491; FDA Registration IRB00004157; Approval letter ID 2021-03-002, date of approval: June 2, 2021). The study was conducted in accordance with the ethical standards as set forth in the Helsinki Declaration of 1975. The study is registered and posted on ClinicalTrials.gov (ClinicalTrials.gov Identifier: NCT05611827).

All participants provided their written informed consent before participation in this study.

#### 2.3.1. Single-Dose Pharmacokinetic Study

On the day of each single-dose intervention, capillary blood samples were taken after a 10 h overnight fast to determine the baseline quercetin concentration (*t* *=* 0). Subsequently, each participant received an oral dose of one of the four interventions described earlier, as well as the standard product. Doses were consumed along with a glass of water (∼200 mL) but without food (4 h fasting post-administration). Following the interventions, capillary whole blood samples were collected at time points 0.5, 1, 2, 3, 4, 6, 8, 10, 12, and 24 h after intervention. A standardized lunch and dinner were served after 4 h and 8 h of product administration, respectively (Figure 1). The lunch consisted of rice noodles with lettuce, pork, chicken, and beef (∼550 calories/serving), and dinner is roast chicken with cabbage salad and whole wheat bread (∼580 calories/serving). All food ingredients were tested by Ultra-High-Performance Liquid Chromatography (UHPLC) for quercetin and related flavonoids to ensure the items would contribute less than 10 mg of flavonoids in the meal.

48 h before each treatment and during the treatment period, participants were instructed to avoid consuming quercetin-containing food such as capers, onions, green tea, or fruits like apples, berries, and grapes or any supplements containing quercetin. A list of quercetin-containing food was provided to study participants.

#### 2.3.2. Multiple-Dose Pharmacokinetic Study

In the multiple-dose phase of the study, participants received the same four interventions (Quercetin LM) daily, for a total of three consecutive days (over a period of 72 h). Capillary blood samples were collected at the same time points as the single-dose phase, except with the addition of collection at 24, 48, and 72 h post-administration: on day one, products were consumed in the same manner as the single-dose phase; on days two and three, products were consumed 1 h prior to blood collection (Figure 1).

The following aspects of the multiple-dose phase were the same as the single-dose phase: sample handling and storage, standardized meals, and food restrictions.

### 2.4. Blood Sample Preparation and Analytical Procedures

On the day of preparation, samples were retrieved from the −20°C freezer and thawed at room temperature. Each 50 *μ*L of sample was mixed with 100 *μ*L of freshly prepared *β*-glucuronidase (*Helix pomatia*, Millipore-Sigma, ≥100,000°IU diluted to 330°IU in pH 5 buffer), incubated at 37 to 40°C for 1 h. 400 *μ*L of acetone was then added for sample extraction, followed by sonication at 40°C for 1 h. 50 mg/L (−)-Epicatechin (Millipore-Sigma, Canada) was included as internal standard for sample analysis. After extraction, samples were centrifuged at 16000 ×g for 5 minutes at room temperature. Supernatants containing quercetin and its metabolites were transferred onto a microplate for LC-MS processing and analyzed using a Thermo Vanquish UHPLC system coupled to a Thermo QExactive Orbitrap Mass Spectrometer. 0.5% formic acid in H_2_O was used as Mobile Phase A, and 0.5% formic acid in acetonitrile was used as B. Liquid chromatography was carried out with a binary solvent gradient progressing from 10% to 100% B in 5 minutes and equilibrated for 3.5 minutes before the next injection. The separation was performed at a flow rate of 400 *μ*L/min on an Acme Xceed *C*_18_, 100 mm × 2.1 mm, 1.9 *μ*m column (Phase Analytical Technology, USA). LC-MS grade solvents and formic acid were obtained from Fisher Scientific (Canada).

The Orbitrap mass spectrometer was calibrated at 70000 resolutions with an accepted range for mass deviation of ±5.0 ppm using Thermo Pierce LTQ Velos ESI Positive Ion Calibration Solution and Thermo Pierce ESI Negative Ion Calibration Solution.

For quercetin, the mass spectrometer was operated in Parallel Reaction Monitoring (PRM) Mode with positive electrospray at a resolution setting of 35000 and a quadrupole isolation window of 1.0 m/z to minimize potential interference from sample matrix. The internal standard (−)-Epicatechin was detected as a hydrogen adduct with a product ion of 139.0388. High resolution mass fragments of quercetin were selected from the top 10 most intense fragments of a previously published accurate mass fragmentation spectrum of quercetin as reported in MassBank of North America (https://mona.fiehnlab.ucdavis.edu/, Accession Number CE000170). The following product ions were extracted from the High-Resolution Mass Spectrometry (HRMS) chromatograms for analysis: 153.0182, 229.0495, 257.0442, 165.0183, and 137.0235. Spectral Similarity Search results from MassBank of North America using a mass accuracy setting of ±20 ppm and filtering for precursor ion of 303.05 returned only quercetin as matching these criteria.

For quercetin metabolites, the mass spectrometer was operated in targeted Selected Ion Monitoring (tSIM) Mode with positive electrospray at a resolution setting of 35,000 and a quadrupole isolation window of 5.0 m/z. Based on previously reported accurate mass data by Lee et al. [25], signal for quercetin is extracted at m/z = 303.0499, that for methylated quercetin is extracted at m/z = 317.0656, that for sulfated quercetin is extracted at m/z = 383.0067, that for quercetin-glutathione conjugate is extracted at m/z = 608.1181, and that for quercetin-N-acetylcysteine conjugate is extracted at m/z = 464.0646. Data were collected using Thermo Xcalibur 5.0 and analyzed in Thermo TraceFinder 5.0 software with default mass tolerance of 5.00 ppm. Quercetin dihydrate (Millipore-Sigma) was used as the chemical standard using a 6-point calibration curve (*R*^2^ > 0.996), and the concentrations of quercetin in capillary whole blood were determined based on internal standard calibration with relative quantification of each quercetin metabolite calculated to quercetin. The test method has an accuracy of 1.6% and precision of 19% CV (coefficient of variation) at LLOQ (Lower Limit of Quantitation) and an accuracy of 3.2% and precision of 13% at ULOQ (Upper Limit of Quantitation).

### 2.5. Data Analysis

The following pharmacokinetic parameters were evaluated: the time to reach peak blood concentration (*t*_max_), maximum total quercetin blood concentration (*C*_max_), and area under the total quercetin blood concentration curve from 0 h (administration time) to 24 h (AUC_0–24_). AUC represents the proportion of the dose that has entered blood circulation. *C*_max_ is the peak blood quercetin concentration following the initial administration of a dose. Pharmacokinetic parameters were calculated using Microsoft Excel 2016. Area under the curve (AUC) was calculated using the trapezoidal method.

As for statistical analysis, comparison of blood concentrations between the tested formulations was performed by ANOVA (Analysis of Variance, general linear model, univariate) followed by post hoc tests for pairwise comparison (Bonferroni Correction). Prior to the statistical tests, normality was assessed using a Shapiro–Wilk test for all dependent variables; non-normal data were log transformed.

Data were considered significant at *p* < 0.05. Results are expressed as mean ± standard deviation (SD). Statistical analyses were completed using Microsoft Excel and IBM SPSS Statistics for Windows (USA, IBM Corp., Version 28.0.1.1); provided figures were developed using GraphPad (CA, USA). As for the power calculation—pre-trials were performed as an adaptive design to (re-)estimate the sample size for the actual trial (dropout rate: 0%), with the statistic power set at 0.05 and 90%. Assuming a dropout rate of 0%, the sample size for the current study was determined to be ten participants. Based on the result of AUC values, power analysis was performed with an effect size of 1.26, power of 1, and significant level of 0.05. Power analysis was performed using the Real Statistics Resource Pack software (Release 7.6).

## 3. Results

All data reported are based on *N* = 10 (Table 1). No withdrawal by any subject occurred during the course of the trial. Participants completed all stages of the trial. Treatments were well tolerated; no adverse events or GI intolerances were reported throughout the trial.

### 3.1. Pharmacokinetics of Formulated vs. Unformulated Quercetin

Two quercetin products (unformulated standard and formulated LM) were tested at different doses in healthy participants (*n* = 10) in a single-dose phase, followed by a multiple-dosing phase of LM only, in which the sustained blood concentrations of quercetin were monitored.

**Figure 2:**
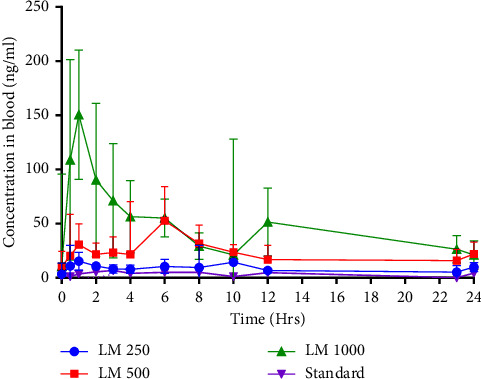
Pharmacokinetic profile of standard vs. quercetin LM. Mean blood concentrations after a single dose of quercetin at different doses of 250 mg, 500 mg, and 1000 mg, over a period of 24 h. Standard product (500 mg) represents unformulated quercetin; the investigational products represent formulated quercetin in LM at different doses of 250 mg, 500 mg, and 1000 mg, respectively. Error bars illustrate ± SD, *n* = 10, for each tested product.

Figure 2 illustrates the mean pharmacokinetic profiles for all four tested quercetin formulations from 0 to 24 h after the oral administration of a single dose (250, 500 or 1000 mg).

**Figure 3:**
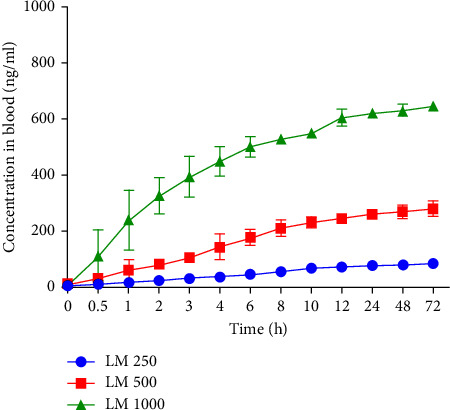
Blood concentrations of quercetin LM over 72 h. Quercetin LM at 500 mg (red) and 1000 mg (green) with significant higher sustained blood concentrations of quercetin compared to quercetin LM (blue). Data are presented as mean ± SD, *n* = 10, for each group.

All pharmacokinetic parameters are summarized in Table 2. The pharmacokinetics such as AUC and *C*_max_ were significantly different between the products (ANOVA, *p* < 0.001, eta-sq = 0.518 and 0.411, respectively). The results of the post hoc tests (Bonferroni) indicated a statistically significant difference between the investigational LM 500 and 1000 and standard product (*p* < 0.001, eta-sq = 0.383; Table 2); however, there was no significant difference between LM 250 and the standard product (AUC_0–24_*p*=0.914, eta-sq = 1.74 × 10^−05^, and *C*_max_*p*=0.759, eta-sq = 0.019; Table 2). The median *t*_max_ values varied between the different treatments from approximately 1 to 6 h.  Figure 3 and Table 3 present the blood concentrations of formulated quercetin over a time of 72 h, after the repeated administration of an oral dose of 250, 500, or 1000 mg LM.

### 3.2. Comparison of the AUC and *C*_max_ Values of the Tested Formulations

Significantly higher concentrations over 24 h were observed for LM as compared to standard quercetin when tested at the same dosage of 500 mg (AUC_0–24_ ratio = 7, *C*_max_ ratio = 7.7; *p* < 0.001, eta-sq = 0.383 and 0.215, respectively; Table 4). LM tested at a 2-fold higher dose (1000 mg) than standard quercetin led to considerable increases in blood concentrations such as 15- and 22-fold higher AUC and *C*_max_ values (*p* < 0.001, eta-sq = 0.46 and 0.379, respectively; Table 2), including approx. 3 times faster peak concentrations, over 24 h (*p* < 0.001, eta-sq = 0.11; Table 2). Conversely, a 2-fold lower dose of LM (250 mg) achieved approx. 3 times greater blood concentrations (AUC_0–24_ ratio = 2.5; *p*=0.914, eta-sq = 1.74*E* − 05; Table 4) and 2-fold higher *C*_max_ values (*C*_max_ ratio = 2.3; *p*=0.759, eta-sq = 0.019; Table 4) than standard quercetin; however, no statistically significant difference was observed in the post hoc analysis.

### 3.3. Circulating Metabolites of Quercetin

The quercetin conjugates in blood were detected following the periodic ingestion of LM 250 mg, over 72 h. Blood samples from participants were treated with beta-glucuronidase during sample preparation, to hydrolyze the glucuronides and determine free quercetin aglycone. Previous studies have reported the covalent binding of quercetin and its metabolites to serum albumin [26–30]. Therefore, beta-glucuronidase treatment of blood samples has been reported as an effective method to release bound quercetin from albumin and provide a consistent estimate of the amount of quercetin and its metabolites in the blood [31, 32]. This means that quercetin glucuronides would also be hydrolyzed, and thus the determination of their concentrations could not be included in the current study. Therefore, the current study focuses on metabolites that are unaffected by the glucuronidase treatment. The major metabolites found in this study were methylated conjugates of quercetin (3′-*O*-methyl and 4′-*O*-methyl quercetin) along with sulfate and glutathione (GSH) conjugates.

Significant differences in concentrations between quercetin conjugates (sulfate vs. methyl vs. GSH) were observed (*p* < 0.001, eta-sq = 0.647; Table 5).

**Figure 4:**
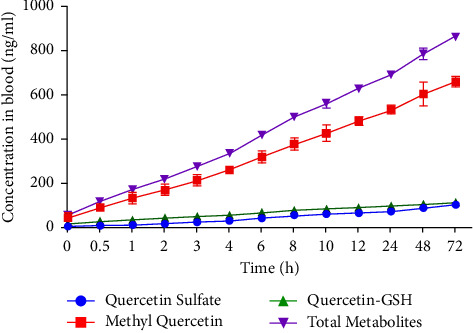
Quercetin metabolite concentrations after oral consumption of LM 250 mg, over a period of 72 h. Error bars illustrate ± SD, *n* *=* 10. Total metabolites present the sum of quercetin sulfate, methyl, and glutathione (GSH) conjugates.

Post hoc analysis (Bonferroni) showed no significant difference between quercetin sulfate and quercetin GSH conjugates (*p*=0.635, eta-sq = 0.004, and *p*=0.963, eta-sq = 0.005, respectively; Table 5).  Figure 4 highlights the mean blood concentrations of quercetin metabolites over 72 h.

## 4. Discussion

One aim of this study was to investigate whether a food-grade delivery formulation of quercetin LM would achieve higher blood concentrations of quercetin than a regular standard formulation. A secondary objective was to determine whether LM could produce sustained higher concentrations of quercetin in the blood after multiple orally administered doses over a 72 h period.

In this trial using a small population (*N* = 10), a new formulation of quercetin, encapsulated in a LM delivery system, has been proven to outperform standard/unformulated quercetin with respect to the pharmacokinetics such as the maximal concentration *C*_max_ and the area under the curve AUC (Figure 2), providing higher sustained concentrations of quercetin over 72 h—accepting the hypothesis of this study.

In the first phase of the study, quercetin blood concentrations were monitored in healthy participants over a 24 h period, after the administration of a single-dose standard (unformulated) and/or formulated quercetin LM. The LM treatments were tested at different concentrations ranging from 250 to 1000 mg. Based on the results, formulated quercetin LM achieved up to 7 times better absorption (703%) than standard quercetin, when tested at the same dose (AUC_0–24_ ratio = 7.0; Table 4). LM administered at a double dose of standard quercetin (1000 mg vs. 500 mg) achieved approximately 15 times higher absorption over 24 h (AUC_0–24_ ratio = 14.6 (1460%); Table 4). Even a lower dose of encapsulated quercetin (LM 250 mg) reached higher concentrations than a 2-fold higher dose of standard quercetin (AUC_0–24_ ratio = 2.6 (255%)). Furthermore, similar patterns of secondary peaks for the quercetin treatments at later time points (around 10 or 12 h) were notable. This might be associated with enterohepatic circulation and re-absorption of quercetin after later food intake when biliary secretion is stimulated. Walle et al. reported that quercetin remains in the organism for an extended period (20–72 h) after ingestion, most likely due to its enterohepatic recycling [33]. The LM 1000 group showed a similar peak at 6 hours, close to the *C*_max_ level observed in LM 500, and a second visible peak occurred at 12 hours, which might be caused by a second round of hepatic cycling at this higher quercetin dose.

The second phase of the study, which was a pilot study approach, specifically focused on the effects of formulated quercetin over a 72 h period, after multiple doses of quercetin LM at concentrations from 250 to 1000 mg (Figure 3). Sustained blood concentrations of quercetin were achieved for each LM treatment.

It is worth noting that relatively high standard deviations in the pharmacokinetics, e.g., *C*_max_ and *T*_max_, were observed within the treatment groups indicating differences in the quercetin absorption by the individual participants, which is in accordance with findings in previous human studies [34–36]. Given the complexity of the pathways of metabolism, these inter-individual variations in quercetin absorption can arise from numerous physiological factors. For instance, this can include age, sex, body mass index (BMI), genetic phenotype, the health of the microbiome/gut microbiota, and gastrointestinal tract, plus dietary history. Also, quercetin absorption in the body is characterized by a high inter-subject variability likely due to variations in intestinal and hepatic phase II quercetin-metabolizing enzymes and transporters which may account for some variability [34–37]. Furthermore, the individual plasma vitamin C status may be associated with inter-individual variations in quercetin concentrations as suggested in a study of Guo et al. [38].

In contrast to other pharmacokinetic studies [38–40], the current intervention was conducted under fasting conditions over a 4 h period in order to minimize possible interactions of the treatments with food compounds such as fiber and fat. Especially the intake of quercetin together with a meal could improve the absorption notably—as a greater surface area promotes dissolution in the intestinal lumen and thus bioavailability [41]. Dietary fat compounds can greatly contribute to quercetin's bioavailability [42] and may even boost the effects of carrier systems like LM by enhancing the micellization. However, human studies studying the impact of dietary factors on the bioavailability of quercetin are still sparse.

Another interesting aspect for studying the bioavailability of quercetin is to determine the circulating metabolites in human blood and thus gain insight into the metabolism of the parent compound and potentially observe a correlation to bioefficacy [17]. Studies suggest that quercetin is mainly absorbed in the upper segment of small intestine and metabolized in the small intestine, colon, liver, and kidney after absorption [43]. The metabolites formed by biotransformation enzymes include methylated, sulfo-substituted, and glucuronidated conjugates [43, 44]. The greatest accumulation of quercetin and its metabolites was found in the lung, liver, and kidney in *in vivo* animal studies [45]. In the present study, the major metabolites identified in the blood, following the administration of LM, were methylated conjugates (3′-*O*-methyl and 4′-*O*-methyl quercetin) along with sulfate and glutathione (GSH) conjugates. Glucuronidated forms were not detected since blood samples were hydrolyzed by beta-glucuronidase before analysis. This has been done because previous studies reported the covalent binding of quercetin and its metabolites to serum albumin [26–30]. Beta-glucuronidase treatment is considered an effective method to release bound quercetin from albumin and thus provide a consistent estimate of the amount of quercetin and its metabolites in the blood [31, 32]. These data on quercetin LM conjugates may be significant for enhancing the biological effects such as the antioxidant properties of quercetin and its conjugated derivatives in the body. For example, a study of Morand et al. showed that the circulating metabolites (e.g., glucuronide and/or sulfo-conjugates) of quercetin possess antioxidant properties based on the results from *in vivo* experiments performed on blood from rats [46]. In the study, the total antioxidant status in rats, when fed a quercetin diet, was higher than the total antioxidant status of the control blood/diet. Furthermore, the circulating derivatives were also found to inhibit low-density lipoprotein (LDL) oxidation and thus may contribute to the antioxidant effects in the blood [46].

Some possible reasons for quercetin's low absorption and oral bioavailability are its premature degradation in the stomach, its poor water solubility (0.01 mg/ml) [47], and low stability of the aglycone (depends, e.g., on the temperature, pH, presence of metal ions, and glutathione). In a previous study, quercetin formulated in LM proved to be more stable in gastric conditions and more soluble in intestinal *in vitro* conditions than unformulated quercetin [39]. Similar results were obtained for another food-grade delivery system, called Phytosome [40]. In another solubility study performed in rats, phospholipid complexation of quercetin could improve the water solubility of the compound (approx. 13-fold) and increase bioavailability such as the AUC and *C*_max_ (3 to 4-fold) when compared to free quercetin [48].

Most recently, the role of intestinal microbiota in the bioavailability and physiological function of dietary polyphenols such as quercetin has been discussed [49, 50]. A study of Di Pede et al. investigated the effects of Quercetin Phytosome®—a similar formulation to LM—on human microbiota [50]. Results demonstrated that quercetin in Phytosome was more stable to microbial degradation than unformulated quercetin, after interaction with the intestinal microbiota.

This might be an important finding, since slowing down the intestinal microbial degradation of quercetin could allow for better absorption of the single compound.

The LipoMicel® delivery system involves the encapsulation of quercetin from *Sophora japonica* L. together with phospholipids that possess dual solubility and therefore act as an emulsifier. Quercetin is released from LM into the lipid environment of enterocyte cell membranes, allowing it to enter the circulation and ultimately reach the targeted site of action in the tissue. LM can be used as an efficient carrier system of various active phytoconstituents to enhance their bioavailability and thus biological activity. One important feature of the presented formulation is that it is composed of natural food-grade ingredients instead of organic solvents and thereby minimizes toxic effects of the carrier material.

Another strength of this study is that the intervention was done in a diet-controlled crossover design on the days when the pharmacokinetic study was conducted. In contrast to other pharmacokinetic studies on quercetin [39, 40], this time the blood concentrations of quercetin, when formulated in a delivery system, were followed over 72 h within a multiple-dose phase, tested at three different doses with a maximal dose of 1000 mg administered. However, since this was a first-time pilot study approach, the design has several limitations. For example, the multiple-dose study phase could have followed a more complete blood collection regimen (e.g., a 24 h pharmacokinetic profile) similar to the single-dose phase. Moreover, a longer observation period (>72 h) might be beneficial for monitoring the sustained quercetin blood concentrations in a multiple-dose study. Furthermore, this work only included a small number of participants; however, the number of subjects in other pharmacokinetic studies on quercetin was usually relatively small [36]. Since actual chemical standards for the metabolites were not available, metabolites were quantified against their parent compound: quercetin. Using actual chemical standards for each metabolite would also improve the reported data.

For future studies, there may be a few interesting approaches to investigate the effects of LipoMicel on improving the oral bioavailability of quercetin.

One such approach might be the influence on tight junctions [51, 52], as well as the interaction with efflux pumps, e.g., through the competition and inhibition of intestinal transporters like the p‐glycoprotein, since these targets represent crucial barriers against paracellular drug absorption and are one of the major contributors to low oral bioavailability of numerous bioactive compounds.

In addition, more studies could examine the influence or contribution of genetic polymorphisms on quercetin metabolism *in vivo*. Also, another objective of future studies may be to demonstrate a correlation between improved uptake and bioavailability of quercetin (such as by LM) and increased efficacy such as positive effects on diverse biomarkers, e.g., cardiometabolic markers.

## 5. Conclusion

Data obtained from this *in vivo* study highlight Quercetin LipoMicel® as a highly efficient formulation that considerably enhances the absorption of quercetin in the body, regardless of the administered dose. Significant increases in blood uptake (255%–1460%) of formulated vs. unformulated quercetin were observed in healthy participants. Sustained higher blood concentrations of quercetin were attained over 72 h after multiple doses of LM.

LM treatments were well tolerated, and no notable side effects were reported. The findings of this work are encouraging and suggest that supplementation with Quercetin LM is a promising strategy to increase the absorption of quercetin in the studied population; however, further research is warranted to consolidate the observations made here.

## Figures and Tables

**Table 1:** Study population demographic data (*N* = 10).

	Mean	SD
Age (years)	37.1	11.2
Weight (kg)	62.4	11.2
BMI (kg/m^2^)	22.3	2.5

BMI: body mass index.

**Table 2:** Pharmacokinetics of quercetin blood concentrations for each tested product.

Product	AUC_0–24_ (ng h/ml)	SD	*C*_max_ (ng/ml)	SD	*T*_max_ (h)	SD
Standard 500 mg	77.3	6.1	6.8	9.2	3.0	3.2
LipoMicel 250 mg	197.0	8.0	15.4	22.0	1.0	2.7
LipoMicel 500 mg	543.1	20.3	52.5	51.7	5.9	2.7
LipoMicel 1000 mg	1128.8	52.3	150.4	117.6	0.9	0.2

AUC: the total area from 0-24 h for blood concentrations of quercetin in each product's pharmacokinetic profile h; *C*_max_: maximum blood concentration; *T*_max_: median time to reach *C*_max_; SD: standard deviation; *n* *=* 10; *p* < 0.001 (ANOVA).

**Table 3:** Quercetin concentrations over 72 h.

Concentration (ng/ml)	LM 250 mg	SD	LM 500 mg	SD	LM 1000 mg	SD
Initial	3.1	3.8	9.7	12.1	9.9	5.1
72 h	82.5	7.7	279.1	27.5	646.4	16.8

LM (250, 500, and 1000 mg); *n* = 10; *p* < 0.001 (ANOVA).

**Table 4:** Comparison of the pharmacokinetics in the single-dose study.

Product	AUC_0–24_ ratio	*C*_max_ ratio
Standard vs. LM 250	2.5	2.3
Standard vs. LM 500	7.0	7.7
Standard vs. LM 1000	14.6	22.1

Quercetin products: standard (500 mg) and LM (250, 500, and 1000 mg) tested in single-dose phase (AUC and *C*_max_ 0–24 h); *n* = 10; *p* < 0.001 (ANOVA).

**Table 5:** Blood concentrations of quercetin metabolites.

Concentration (ng/ml)	Quercetin sulfate	SD	Methyl quercetin	SD	Quercetin GSH	SD	Total metabolites	SD
Initial	0.5	1.0	40.3	19.7	10.4	6.3	51.2	9.7
72 h	98.0	11.6	657.1	23.7	106.0	4.3	861.5	9.9

Reported data are the mean concentrations of different metabolites following the administration of LM 250 mg over 72 h; *n* = 10; *p* < 0.001 (ANOVA). Total metabolites present the sum of quercetin (Q) sulfate, methyl, and glutathione (GSH) conjugates.

## Data Availability

The data used to support the findings of this study are available from the corresponding author upon request.
